# Design and Validation of Low-Cost, Portable Impedance Analyzer System for Biopotential Electrode Evaluation and Skin/Electrode Impedance Measurement

**DOI:** 10.3390/s25123688

**Published:** 2025-06-12

**Authors:** Jaydeep Panchal, Moon Inder Singh, Mandeep Singh, Karmjit Singh Sandha

**Affiliations:** Department of Electrical and Instrumentation Engineering, Thapar Institute of Engineering and Technology, Bhadson Rd, Patiala 147004, Punjab, India; misingh@thapar.edu (M.I.S.); mandeep@thapar.edu (M.S.); kssandha@thapar.edu (K.S.S.)

**Keywords:** impedance analyzer, AD5933, portable, impedance measurement, skin/electrode impedance, biopotential electrode

## Abstract

This paper presents a novel, low-cost, portable impedance analyzer system designed for biopotential electrode evaluation and skin/electrode impedance measurement, critical for enhancing bioelectrical signal quality in healthcare applications. In contrast with conventional systems that depend on external PCs or host devices for data acquisition, visualization, and analysis, this design integrates all functionalities into a single, compact platform powered by the Analog Devices AD5933 impedance converter and a Raspberry Pi 4. The design incorporates custom analog circuitry to extend the measurement range from 10 Hz to 100 kHz and supports a wide impedance spectrum through switchable feedback resistors. Validated against a benchtop impedance analyzer, the system demonstrates high accuracy with normalized root-mean-square errors (NRMSEs) of 1.41% and 3.77% for the impedance magnitude and phase of passive components, respectively, and 1.43% and 1.29% for the biopotential electrode evaluation and skin/electrode impedance measurement. This cost-effective solution, with a total cost of USD 159, addresses the accessibility challenges faced by smaller research labs and healthcare facilities, offering a compact, low-power platform for reliable impedance analysis in biomedical applications.

## 1. Introduction

Electrical impedance, denoted by “Z”, is a fundamental parameter that quantifies the resistance and reactance experienced by an electrical circuit when an alternating current (AC) flows through it. A complex quantity is a complex number comprising both a magnitude and a phase angle, measured in ohms (Ω). Impedance measurement plays a critical role in diverse fields and applications. It provides insights into the electrical properties of materials, making it essential in materials science, corrosion monitoring [1], electrical engineering, and physics for designing and optimizing electronic devices, circuits, and systems. Impedance measurement is also used in biomedical research and healthcare applications, such as cell culture growth monitoring [2], the measurement of skin/electrode impedance, and evaluating the performance of biopotential electrodes used in recording bioelectrical signals in the human body [3,4]. Bioelectrical impedance analysis (BIA) is another important application of impedance measurement in healthcare [5].

Disposable pre-gelled electrocardiogram (ECG) electrodes play a vital role in ECG monitoring within various healthcare settings. These electrodes are designed to be convenient, hygienic, and easy to use. They consist of a conductive gel pre-applied to the electrode surface, eliminating the need for additional gel application during ECG measurement. This gel helps ensure good electrical contact between the skin and the electrode, enabling accurate and reliable ECG signal acquisition. These electrodes are extensively used in intensive care units (ICUs) and critical care units to continuously monitor patients’ heart rate and rhythm [6], which is vital in alerting ICU staff to cardiac issues. They undergo bench tests to ensure the electrodes’ effectiveness and electrical properties. One such test involves measuring the impedance of a pair of electrodes that are directly connected gel to gel. By evaluating the electrical properties through impedance measurements, manufacturers can confirm that the electrodes meet the required standards and provide reliable ECG signals when used in clinical settings [7,8,9].

Skin/electrode impedance refers to the resistance encountered by the electrical signal as it travels through the interface between the electrode and the patient’s skin. It has a significant impact on the measurement of various biopotential signals, including electroencephalogram (EEG), electromyogram (EMG), and electrocardiogram (ECG) signals. The impedance is influenced by factors such as the skin’s condition, the design of the electrode, and the conductivity of the gel or adhesive applied. A low impedance is desirable because it ensures proper electrical contact and reduces signal distortion or noise. Conversely, high impedance can lead to poor signal quality, resulting in artifacts and inaccuracies in the signal recording. Assessing the impedance of the skin/electrode interface is vital before acquiring biopotential signals, especially for EEG measurements [10,11,12,13]. Traditionally, such measurements have relied on bulky benchtop analyzers, limiting their use in portable and point-of-care scenarios. In response to the demand for field-deployable, cost-effective alternatives, significant research has focused on the development of miniaturized and portable impedance analyzers.

A common foundation across many of these systems is the AD5933 impedance converter from Analog Devices, a compact chip integrating a frequency generator and digital signal processing. Several researchers have adapted this IC to construct portable analyzers tailored for biological applications. For instance, Margo et al. implemented a four-electrode configuration to minimize electrode–medium interface artifacts, enhancing the accuracy for small physiological samples [14]. Hoja and Lentka presented a miniaturized family of impedance analyzers using the AD5933 device in conjunction with advanced microcontrollers (AVR32 family) and ZigBee modules for telemetric control [15]. Punter-Villagrasa et al. introduced a point-of-use portable EIS system for biomedical applications, using a custom potentiostat and digital lock-in amplifier [16]. Chabowski et al. highlighted the need for additional signal conditioning and proposed frequency-planning methods to avoid aliasing issues inherent to the AD5933 device’s DDS generator [17]. Similarly, Al-Ali et al. presented a portable analyzer combining the AD5933 device with an ATmega328P MCU and Bluetooth, which faced limitations in its frequency range and calibration accuracy across wide impedance variations [18].

Recent advancements have also focused on applications such as saline solution characterization [19], corrosion monitoring [20], skin bioimpedance for ulcer prediction, in vivo biosensing [21], and fruit quality monitoring in smart agriculture [22]. These implementations, though diverse in application, depend on external PCs for control and data visualization.

This work involved the development and validation of an impedance measurement system designed to measure skin/electrode impedance and evaluate disposable electrodes. The proposed impedance analyzer is highly efficient and has standalone operation and low power consumption, making it an ideal solution for portable and cost-effective impedance measurements. One of the most significant advantages of this development is that it eliminates the need for a personal computer to conduct impedance measurements and visualize the results.

## 2. Impedance Analyzer System

The impedance analyzer system is built on two key components: a Raspberry Pi 4 (**Raspberry Pi Holdings plc, Cambridge, UK**) and a specialized impedance converter board. The Raspberry Pi 4 offers an economical yet powerful solution with a 1.8 GHz quad-core CPU, 4096 MB of RAM, and both WLAN and Bluetooth low-energy capabilities in compact dimensions of 85.6 × 56.5 mm. The custom-designed impedance converter board integrates seamlessly into the Raspberry Pi through a 40-pin header. Central to the impedance converter board is the AD5933 chip from Analog Devices (**Analog Devices, Inc., Wilmington, MA, USA**), specifically engineered to measure and convert electrical impedance. This impedance converter board and the Raspberry Pi communicate efficiently via the I2C protocol, ensuring consistent and rapid data transfer. Together, these components deliver a comprehensive solution for impedance analysis.

### 2.1. AD5933 IC

The AD5933 device is an advanced integrated circuit that provides a highly accurate and complete solution for measuring impedance. It incorporates various components, including an onboard frequency generator, a 12-bit analog-to-digital converter (ADC), and a digital signal-processing (DSP) engine. The frequency generator integrated into the AD5933 device stimulates an external complex impedance at a known frequency. As the impedance reacts to the excitation signal, AD5933’s internal trans-impedance amplifier (TIA) converts the response signal into a corresponding voltage signal. The onboard ADC then samples this voltage signal. The ADC is responsible for capturing the real and imaginary components of the impedance response at each excitation frequency. The built-in DSP engine subsequently processes this captured data. The DSP engine performs a discrete Fourier transform (DFT), which allows the AD5933 device to analyze and extract essential information from the captured data. For every excitation frequency, the DFT engine of the AD5933 device produces real and imaginary data words, which can be accessed through the device’s I2C interface.

### 2.2. Expanding Frequency Range of Measurement

The AD5933 chip has an internal clock source of 16 MHz, which allows impedance measurements in the 1–100 kHz frequency range. However, for frequencies below 1 kHz, an external clock source with different frequencies is required, as shown in Table 1. To address this, the hardware pulse width modulation (PWM) output pin of the Raspberry Pi 4, which serves as the single-board computer in the proposed design, is connected to the master clock (MCLK) pin of the AD5933 device. This arrangement allows a PWM signal with a fifty percent duty cycle and the required frequency to be used as a clock source for the AD5933 device.

### 2.3. Expanding Impedance Range of Measurement

It is worth noting that the excitation signal generated by the internal frequency generator of the AD5933 device contains a DC offset, which can polarize the sample under testing and reduce the dynamic range of the internal ADC. To mitigate this issue and minimize the output impedance of the AD5933 chip, additional circuitry in the form of a high-pass filter and an external trans-impedance amplifier (TIA) is implemented in the impedance converter board. This approach is suggested by the manufacturer’s application note [23]. By incorporating these additional circuitries, the proposed design ensures accurate and reliable impedance measurements for a wide impedance range.

Additionally, the implemented external TIA requires multiple feedback resistors to support various impedance values. To address this requirement, the proposed design of the impedance analyzer includes multiple switchable feedback resistors in the impedance converter board. Two feedback resistors are incorporated into the design, and the AS849 digital single-pole double-throw switch facilitates their selection. This switch allows for the switching between the two feedback resistors depending on the measured impedance value. The control of the AS849 switch is managed by a general-purpose input–output (GPIO) pin of the Raspberry Pi 4, providing a convenient and flexible means of adjusting the feedback resistor configuration based on the analyzed impedance.

### 2.4. Calibration

The AD5933 device must be calibrated using a known impedance to calculate the gain factor. Once the gain factor is determined, it can be used to calculate the impedance and phase of any unknown sample connected between the VOUT and VIN pins of the AD5933 using Equations (1) and (2). The methods described in the AD5933 datasheet [24] are suitable for small frequency ranges, but this study aimed to cover a more comprehensive frequency range. Hence, a calibration process was utilized, employing a lookup table to determine gain factor values for each frequency point in the sweep. In this study, an impedance analyzer system was designed to calculate the magnitude at each sweep frequency by utilizing the values in the real and imaginary registers. This calculation is performed when a known resistor with an impedance magnitude of Z_k_ is connected using Equation (3). The determined gain factors will remain valid if there are no alterations to the TIA feedback resistor, output excitation voltage, or PGA gain.

(1) $$Impedance=\frac{1}{Gain\;Factor\times \sqrt{{Real}^{2}+{Imaginary}^{2}}}$$

(2) $$Phase=\tan^{-1}\frac{Imaginary}{Real}$$

(3) $$Gain\;Factor=\frac{1/Z_{k}}{\sqrt{{Real}^{2}+{Imaginary}^{2}}}$$

### 2.5. Hardware Architecture

**Figure 1:**
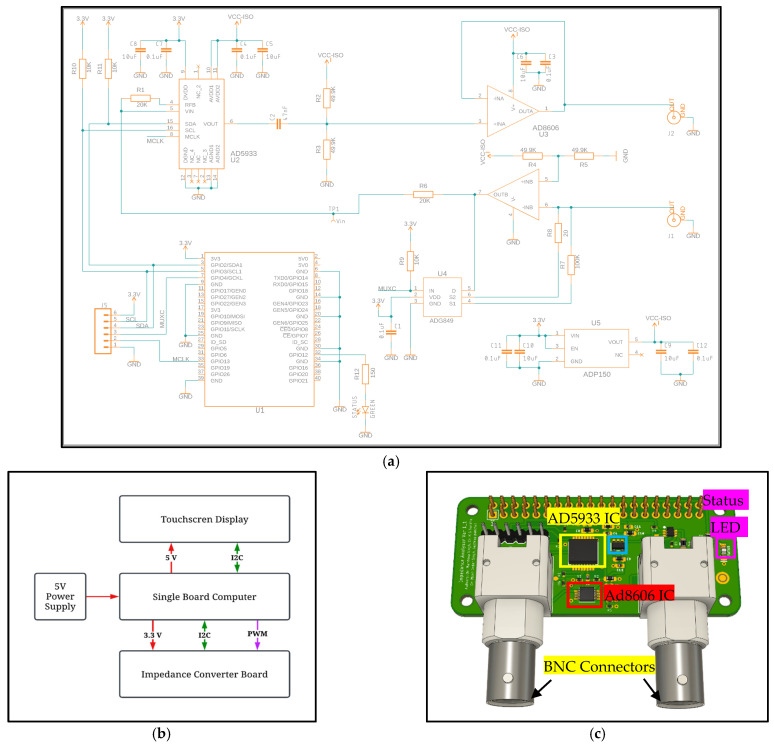
(**a**) Schematics of impedance converter board, (**b**) hardware architecture block diagram, and (**c**) components of impedance converter board.

Figure 1a illustrates the impedance analyzer system’s hardware schematics. The single-board computer receives power from a battery pack or a 5 V supply. The impedance converter board is powered by 3.3 V, while the touchscreen is powered by 5 V sourced from the single-board computer. The master clock for the AD5933 IC is generated directly through the hardware PWM of the single-board computer. Communication between the single-board computer, touchscreen, and impedance converter board occurs via I2C communication. Figure 1b, illustrates the impedance analyzer system’s hardware architecture, and Figure 1c displays the different components of the impedance converter board. These components include the AD5933 device (U2), two operational amplifiers of an AD8606 device (U3), and an ADG849 analog multiplexer (U4). The ADG849 device is utilized to select between the feedback resistors (100 kΩ and 200 Ω). All of the external analog circuitry and the internal analog circuitry of the AD5933 device are powered by a low-noise ADP150 (U5) regulator.

### 2.6. Software Architecture

**Figure 2:**
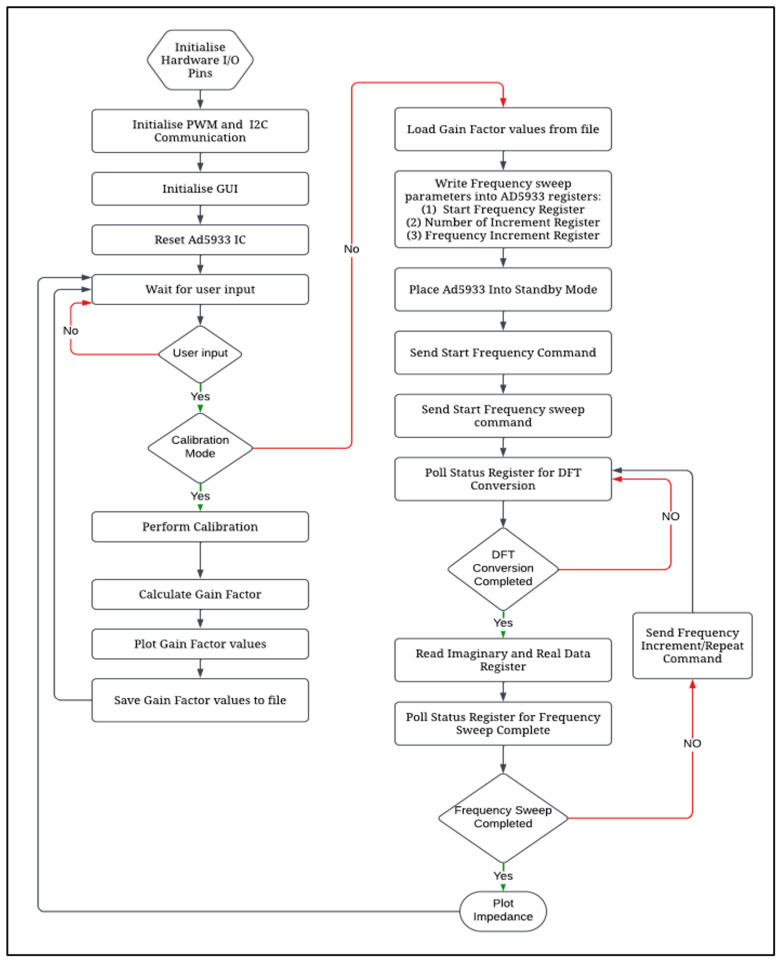
Impedance analyzer system software algorithm.

**Figure 3:**
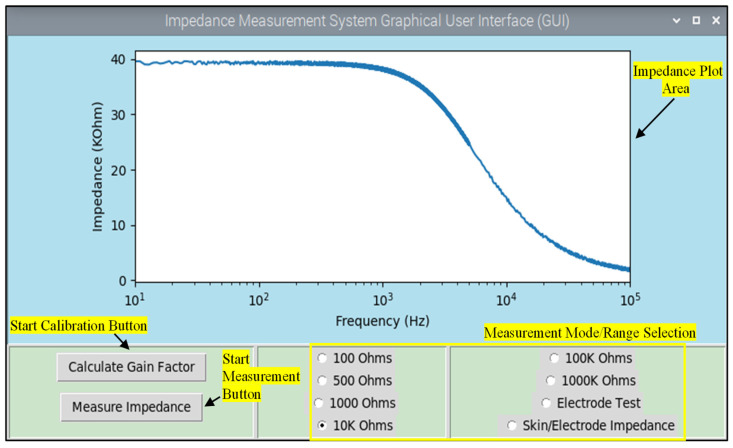
Graphical user interface of impedance analyzer system.

The software code was developed using the Python (V 3.9) programming language, and the Integrated Development and Learning Environment (IDLE) was utilized for the development process. The code is the backbone of the entire system, orchestrating its functionalities in a predefined sequence. An algorithm was developed, as illustrated in Figure 2. The sequence commences with the software’s interaction with the user through a graphical user interface (refer to Figure 3). Furthermore, the software code is responsible for governing the pulse width modulation (PWM) frequency and managing the switching of the resistors during the frequency sweep.

## 3. Results and Discussion

**Figure 4:**
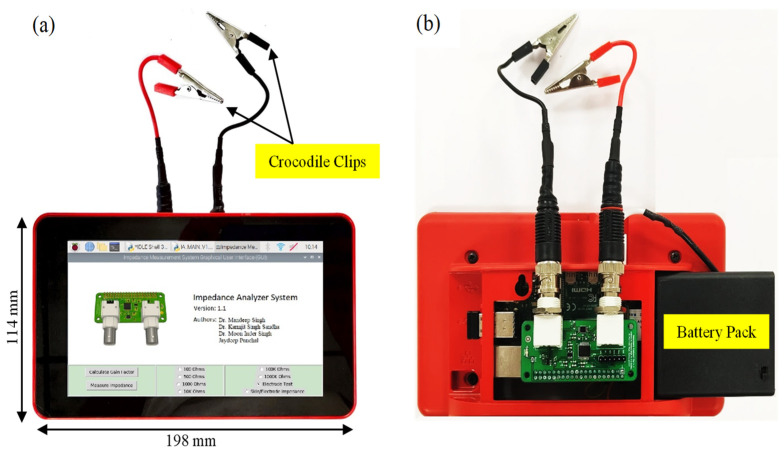
Developed impedance analyzer system prototype: (**a**) front side; (**b**) back side.

The developed impedance analyzer system, as shown in Figure 4, underwent testing in various experiments to assess its performance in different scenarios. Initially, passive components were used to validate the accuracy of the acquired data compared with theoretical circuit values and to evaluate the calibration performance. Additionally, the system’s ability to assess disposable electrodes and measure skin/electrode impedance was examined by comparing the results with those obtained from a benchtop impedance analyzer.

### 3.1. Passive Components Analysis

**Figure 5:**
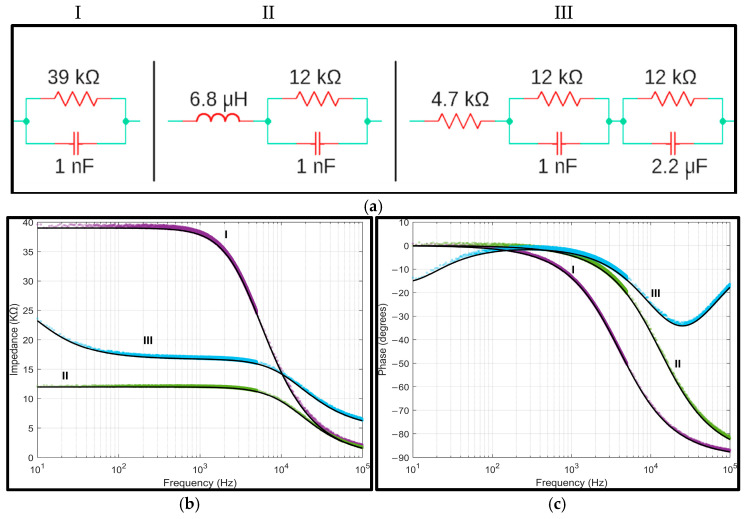
(**a**) Test circuits used for impedance analyzer validation. (**b**) Measured impedance magnitude and (**c**) phase [circuit I (°), circuit II (°), and circuit III (°)] compared with the theoretical values (-).

A preliminary test was conducted using a two-terminal measurement setup in three series–parallel circuits to evaluate the impedance analyzer system’s performance, similar to the method suggested in [22]. These circuits were constructed with various configurations using off-the-shelf components. Figure 5a illustrates the test circuits employed for system validation and the respective component values. Circuit I was a parallel combination of a 39 kΩ resistor and a 1 nF capacitor. Circuit II was formed by placing a 6.8 µH inductor in series with a parallel combination of a 12 kΩ resistor and a 1 nF capacitor. Lastly, circuit III incorporated a series arrangement of a 4.7 kΩ resistor, a parallel combination of a 12 kΩ resistor and a 1 nF capacitor, and another parallel combination of a 12 kΩ resistor and a 2.2 µF capacitor. The Keysight DAQ970A device was utilized to measure the actual values of components employed for testing circuits, as these components possess tolerances. Figure 5b depicts the magnitude and phase values obtained from the impedance analyzer system and the corresponding theoretical curves.

The accuracy of the system was evaluated by calculating the normalized root-mean-square error (NRMSE) and root-mean-square error (RMSE) between the measured data and theoretical calculations across the entire frequency range. To test the repeatability, multiple impedance measurements at different frequencies were performed in each circuit under identical conditions, and the coefficient of variation (CV) for the impedance magnitude and phase was calculated (refer to Table 2).

### 3.2. Disposable Electrode Evaluation

By evaluating the electrical properties of disposable biopotential electrodes through impedance measurements, manufacturers can confirm that the electrodes meet the required standards and provide reliable ECG signals when used in clinical settings. Electrodes are connected gel to gel for electrode evaluations; the impedance is measured at 10 Hz. A low impedance value of lower than 2 kΩ is desirable for better signal transmission and lower motion artifacts or noise that can interfere with the accuracy of the ECG measurements [25].

**Figure 6:**
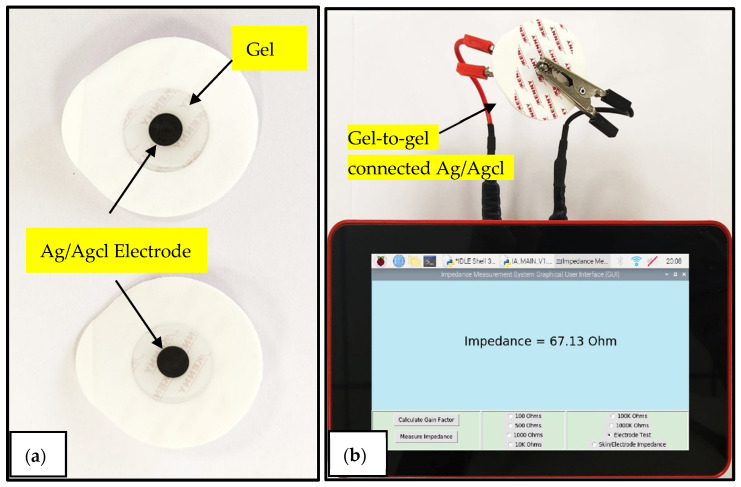
(**a**) Ag/Agcl disposable electrodes; (**b**) two disposable electrodes attached in a gel-to-gel manner connected to the impedance analyzer system.

As shown in Figure 6, commercially available disposable electrodes from two different manufacturers, Romsons and 3M, were connected gel to gel, and their impedance was measured at a 10 Hz frequency. The graphical user interface provides a dedicated option to perform electrode evaluation tasks. In this study, 12 pairs of Ag/Agcl disposable electrodes connected in a gel-to-gel manner were tested, and their impedance values were compared with the benchtop impedance analyzer Fluke PM6306 as a reference instrument (refer to Table 3). The impedance analyzer system had a root-mean-square error of 0.5088 ohms and a normalized root-mean-square error of 1.43% compared with the reference instrument.

### 3.3. Skin/Electrode Impedance Measurement

The skin/electrode impedance plays a crucial role in the quality of EEG signals. When the skin/electrode impedance is high, it can cause signal attenuation, distortion, and higher noise levels. Since EEG signals are inherently weak, they are susceptible to interference from various factors during measurement, leading to artifacts that adversely impact signal quality. In order to obtain reliable EEG signals, it is crucial to keep the impedance between the electrodes below 10 kΩ [26]. It is required to perform skin preparation, which entails shaving, abrading, and cleaning the skin surface to achieve low skin/electrode impedance levels. Following the skin preparation, a conductive gel is applied to the skin, which hydrates the upper layer and reduces the impedance [27].

**Figure 7:**
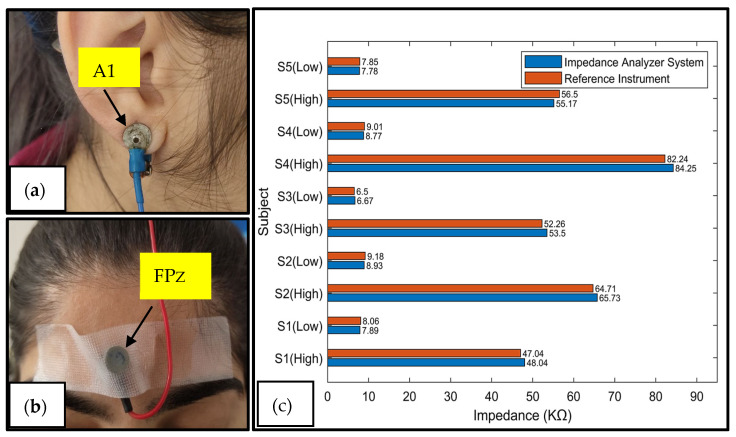
(**a**) Reusable Ag/Agcl electrode connected to A1, (**b**) reusable Ag/Agcl electrode connected to FPZ, and (**c**) comparison of impedance measurements from reference instrument and developed impedance analyzer system for skin/electrode impedance measurements.

In this study, the researchers measured the skin/electrode impedance between two specific EEG electrode placement sites, A1 and FPz, of the 10–20 electrode placement system. A1 refers to the electrode placed on the left earlobe, commonly used as a reference electrode due to its minimal electrical activity and stable contact with the skin. FPz denotes the frontal pole midline, located at the center of the forehead. The choice of A1 and FPz was motivated by their common use in clinical EEG setups, where A1 serves as a stable reference point and FPz provides a practical site for impedance testing due to its minimal hair interference and ease of electrode application. The impedance was measured at 25 Hz, similar to a commercially available skin/electrode measurement system [28]. This electrode placement is depicted in Figure 7a,b. Measurements were taken before and after the skin preparation and application of the conductive gel to assess the impedance levels. The impedance measurements were conducted on five subjects using an impedance analyzer system and a benchtop reference instrument. Figure 7c compares the results of the two measurement methods. Before the skin preparation and gel application, the impedance values are denoted as Sx (high). In contrast, the impedance values after the skin preparation and gel application are denoted as Sx (low). The root-mean-square error was 978 ohms, and the normalized root-mean-square error was 1.29% compared with the reference instrument. This comparison provided insights into the accuracy and reliability of the impedance analyzer system in measuring the skin/electrode impedance before and after skin preparation and gel application.

### 3.4. Power Consumption

**Figure 8:**
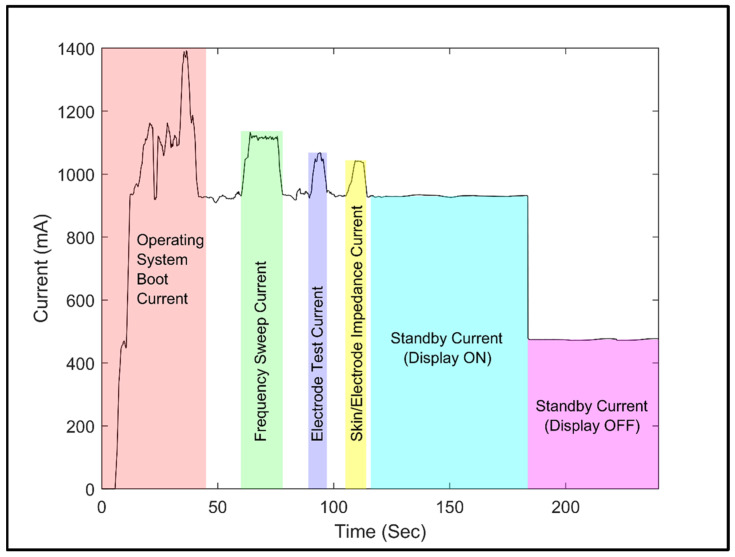
Current consumption of impedance analyzer system.

In Figure 8, we can observe the current consumption of the impedance analyzer system under different operating conditions. The data were collected using a Keysight DAQ970A (**Keysight Technologies, Inc., Santa Rosa, CA, USA**) device with the DAQM901A current-sensing module. During the frequency sweep operations, the impedance analyzer system maintained an average power consumption of 5.42 W. The average power consumption for the electrode testing was 5.06 W, while for the skin/electrode impedance measurements, it was 5.00 W. In standby mode, with the display on, the single-board computer consumed an average power of 4.64 W, whereas with the display off, it consumed 2.38 W. The booting process required an average power of 4.23 W.

### 3.5. Cost Analysis and Comparison

Table 4 provides a cost analysis to classify the device developed in this study as low-cost. Throughout this document, all prices are denoted in USD for ease of reference.

Table 5 compares the performance of the developed impedance analyzer system and similar systems described in the existing literature. The system developed in this study demonstrated a close frequency range, accuracy, and excitation voltage compared with similar instruments. This similarity can be attributed to the utilization of the AD5933 chipset. Notably, the maximum error in the impedance magnitude was 1.43%, slightly higher than the 1.20% reported in [22]. However, the maximum phase error of 3.77% aligns with the performance of most considered instruments. One major drawback of previously developed systems is their reliance on a PC or smartphone connected via USB or Bluetooth for acquiring, visualizing, and storing impedance data. In contrast, our impedance analyzer system allows for the visualization and storage of measurements within its internal memory, making it completely standalone. The combination of these features, along with the Python-based firmware, creates a flexible, cost-effective, and highly portable system. The weight of the developed impedance analyzer system is approximately 880 g.

## 4. Conclusions

This paper presents the design, testing, and validation of a portable impedance analyzer system for disposable biopotential electrode evaluation and skin/electrode impedance measurements. The system is based on the AD5933 device and operates between 10 Hz and 100 kHz. It incorporates internal selectable feedback resistors and is designed to be compact. The system demonstrates a good level of accuracy compared with a benchtop impedance analyzer for both passive electrical components and when testing biopotential electrodes and measuring skin/electrode impedance. Its accuracy, portability, standalone operation, low cost, and user-friendly nature, along with its firmware customizability, make it suitable for various applications, including biopotential electrode evaluation, skin/electrode impedance measurement, and other impedance measurement tasks.

## Figures and Tables

**Table 1:** External clock frequencies required to support wide frequency range.

Frequency Range of Measurement	Required External Clock Frequency
1 kHz to 300 Hz	2 MHz
300 Hz to 200 Hz	1 MHz
200 Hz to 100 Hz	250 kHz
100 Hz to 30 Hz	100 kHz
30 Hz to 20 Hz	50 kHz
20 Hz to 10 Hz	25 kHz

**Table 2:** Accuracy of impedance analyzer system.

	Magnitude			Phase		
Circuit	RMSE (Ω)	CV (%)	NRMSE (%)	RMSE (°)	CV (%)	NRMSE (%)
I	379.3	0.72	1.01	2.96	1.21	3.7
II	174.5	0.81	1.67	3.28	1.42	4.1
III	262.2	0.53	1.54	2.8	1.14	3.5
**Average**	**272**	**0.69**	**1.41**	**3.01**	**1.26**	**3.77**

**Table 3:** Impedance measurement comparison of 12 pairs of Ag/Agcl disposable electrodes.

Electrode Pair	P1	P2	P3	P4	P5	P6	P7	P8	P9	P10	P11	P12
**Impedance analyzer system**	69.72 Ohm	77.91 Ohm	64.2 Ohm	100.28 Ohm	63.37 Ohm	64.32 Ohm	64.92 Ohm	68.76 Ohm	68.8 Ohm	67.13 Ohm	75.57 Ohm	76.31 Ohm
**Reference instrument (Fluke PM6306)**	69.24 Ohm	78.01 Ohm	64.36 Ohm	99.82 Ohm	64.35 Ohm	64.61 Ohm	65.34 Ohm	69.35 Ohm	68.39 Ohm	66.78 Ohm	74.96 Ohm	75.68 Ohm

**Table 4:** Cost analysis of impedance analyzer system.

Component	Cost (USD)
Single-board computer	44
Touchscreen display	61
Ad5933 IC	22
PCB	12
Other passive/active components	20
**Total cost**	**159**

**Table 5:** Comparison of portable impedance analyzers.

Reference	Frequency Range	Excitation Voltage	Internal Feedback Resistors	Maximum Error	External Device Required	Cost (USD)
[14]	1–100 kHz	20–200 mV	No	2%—magnitude; 1.5°—phase	Yes	-
[15]	0.01–100 kHz	1 mV–1 V	Yes	1.6%—magnitude; 0.6°—phase	Yes	-
[16]	10–100 kHz	10 Vrms	Yes	12.3%—magnitude; 12.1°—phase	Yes	-
[17]	1–100 kHz	200 mV, 400 mV, 1 V, and 2 V	Yes	3.5%—magnitude; 2.8°—phase	Yes	-
[18]	5–100 kHz	200 mV, 400 mV, 1 V, and 2 V	Yes	N/A	Yes	150
[19]	10–100 kHz	100 mV, 500 mV, and 1 V	Yes	N/A	Yes	100
[20]	0.01–100 kHz	10 mV–2 V	No	5%—magnitude; 3°—phase	Yes	167
[21]	5–100 kHz	25 mV, 50 mV, 200 mV, 400 mV, 1 V, 2 V, and 3 V	Yes	6%—magnitude; 3°—phase	Yes	-
[22]	10–100 kHz	200 mV, 400 mV, 1 V, and 2 V	Yes	1.2%—magnitude; 3.8°—phase	Yes	-
Keysight E4990A (**Keysight Technologies, Inc., Santa Rosa, CA, USA**)	20 Hz–120 MHz	Variable (model-dependent)	N/A	<1%—magnitude; <1°—phase	No	20,000–30,000
Fluke PM6306 (**Fluke Corporation, Everett, WA, USA**)	50 Hz–1 MHz	Variable (model-dependent)	N/A	<1%—magnitude; <1°—phase	No	5000–7000
Hioki IM3570 (**HIOKI E.E. CORPORATION, Ueda, Japan**)	4 Hz–5 MHz	Variable (model-dependent)	N/A	<1%—magnitude; <1°—phase	No	8000–12,000
Proposed work	10–100 kHz	200 mV, 400 mV, 1 V, and 2 V	Yes	1.43%—magnitude; 3.77°—phase	No	159

## Data Availability

The raw data supporting the conclusions of this article will be made available by the authors on request.
